# Potentials, barriers, and strategies for integrating tuberculosis, diabetes mellitus, and hypertension case management: A scoping review

**DOI:** 10.1371/journal.pone.0345708

**Published:** 2026-06-26

**Authors:** Ari Probandari, Kyra Modesty, Vivienne Tjung, Brigitta Elycia Sitepu, Febby Gunawan Siswanto, Kerub Dion, Victoria Sari, Ailiana Santosa, Nawi Ng, Vitri Widyaningsih

**Affiliations:** 1 Disease Control Research Group, Faculty of Medicine, Universitas Sebelas Maret, Surakarta, Indonesia; 2 Department of Public Health and Preventive Medicine, Faculty of Medicine, Universitas Sebelas Maret, Surakarta, Indonesia; 3 School of Public Health and Community Medicine, Institute of Medicine, Sahlgrenska Academy, University of Gothenburg, Gothenburg, Sweden; North-West University, SOUTH AFRICA

## Abstract

**Background:**

The coexistence of tuberculosis (TB) with other chronic diseases, such as diabetes mellitus (DM) and hypertension, presents a growing challenge for efforts to end TB due to the complex interactions of coordinated care among relevant care providers and increased risk of adverse outcomes. The comorbidities between TB and DM underscore the necessity of integrated care approaches that span screening, diagnosis, and treatment. Although integrated care models have the potential to improve patient outcomes by supporting people to complete treatment, improving retention in care, and streamlining service delivery, the understanding of low integration within existing health systems is also limited. This scoping review aims to map existing models of integrated TB, DM, and hypertension case management, identify potential benefits and examine barriers to integration, and define strategies for effective implementation.

**Methods:**

This scoping review examined original research papers on the integrated management of TB, DM, and hypertension, published from January 2005 to January 2025. Studies of various designs were included and sourced from databases such as MEDLINE, Scopus, ScienceDirect, and EMERALD using targeted search terms. Four reviewers independently screened and extracted data using a standardized form. Findings were synthesized qualitatively and discussed with experts for additional insights.

**Discussion:**

From 7,983 studies screened, 126 studies met the inclusion criteria. The findings reveal that integrated management of TB, DM, and hypertension can improve access to care, program retention, and early detection of comorbidities. Integration of services—including screening, diagnosis, treatment, counseling, and support for patients’ self-management—was generally well-received, practical to implement, and contributed to improved patient outcomes. Nevertheless, several barriers remain, such as fragmented health systems, lack of standardized protocols, inadequate provider training, limited health information systems, and insufficient financing mechanisms. Addressing these challenges requires systemic interventions, including strengthened policy and regulatory frameworks, capacity-building through structured training, robust and interoperable information systems, inter-program coordination, task-shifting strategies, and patient-centered care approaches. While the evidence highlights the potential of integrated care, gaps remain in demonstrating long-term outcomes and cost-effectiveness, underscoring the need for further research and evaluation to support the scale-up of successful models across diverse health system contexts.

## Introduction

The health burden in low- and middle-income countries (LMICs) has shifted from infectious diseases to non-communicable diseases (NCDs). The World Health Organization (WHO) estimated that two-thirds of hypertension cases [1] and 3 in 4 adults with diabetes would be found in LMICs in 2025 [2]. The coexistence of both conditions is particularly concerning because high blood sugar levels can damage blood vessels and increase blood pressure, while hypertension can further complicate diabetes management and increase the risk of cardiovascular complications [3]. Despite the escalating burden of NCDs, the prevalence of epidemics remains high. Tuberculosis (TB) continues to exist in LMICs, contributing to their high mortality rates [4,5]. The combination of TB and NCDs offers a different level of challenges. TB patients with DM tend to present with higher bacillary loads compared to their non-diabetic counterparts [6].

On the other hand, health systems in developing countries often operate in silos. Lack of data quality and equity results in inadequate data-driven decision-making, which causes longer health service delivery and poor health outcomes [7]. On top of that, governance, the health system, and financial support are also crucial to achieve greater integrative health management [8]. Despite the individuality of health systems in developing countries, the dual burdens of infectious and NCDs in LMICs underscore the need for integrated disease management by involving screening, diagnosis, treatment, and care for all three health conditions [9]. There is still limited review on the potential and barriers in the integration of TB, DM, and hypertension management, particularly in LMICs. This review aimed to map the concepts of integrated disease management and health service integration while also identifying the potential, barriers, and key strategies associated with the integrated management of TB, DM, and hypertension.

## Materials and methods

We conducted a scoping review following the framework outlined by Arksey and O’Malley enhanced by Leval et al. [10], which consists of five steps: (1) identifying the research question, (2) identifying relevant studies, (3) study selection, (4) charting the data, and (5) collating, summarizing, and reporting results, and conducting consultation. Details of population, concept, and context are described in the previous publication [11]. The review was reported in accordance with the Preferred Reporting Items for Systematic Reviews and Meta-Analyses Extension for Scoping Reviews (PRISMA-ScR), and the completed PRISMA-ScR checklist is provided as Supporting Information (S1 File).

This scoping review is part of the Integrated Model for Tuberculosis, Diabetes, and Hypertension Screening Among Workers (INSIGHT) Project, which has obtained ethical clearance No. 09/UN27.06.6.1/KEP/EC/2021. The scoping review is not subject to obtaining informed consent, as it does not involve the collection or use of data from human participants.

### Identification of research questions

We searched for all original research papers relevant to TB, DM, and hypertension management. We searched literature using Medical Literature Analysis and Retrieval System Online (MEDLINE), Scopus, ScienceDirect, and EMERALD, as well as a Google search for grey literature. Keywords used in this study included ‘tuberculosis’, ‘diabetes mellitus’, ‘hypertension’, ‘integration’, ‘diagnosis’, ‘treatment’, and ‘screening’. The Boolean Logic (AND and OR) and keywords for searching literature relevant to the integration of TB, DM, and hypertension management used in this review article followed the previous protocols published [11]. On the other hand, the OR operator is applied to broaden the scope of a condition, returning results that meet at least one of the specified criteria from “tuberculosis” OR “TB”.

### Selection of studies

We selected studies published from January 2005 to January 2025 in English. Four reviewers (KM, FGS, BES, and KD) independently screened the titles and abstracts of the studies and conducted a full-text review of selected studies, independently extracting relevant data. However, despite any disagreements, AP and VW checked all the results of screening by these reviewers. Disagreements among reviewers were discussed and resolved by other researchers (AP and VW). The selected studies were synthesized qualitatively by grouping them into several themes, including the concept of integration and its level of implementation within health systems. The results were discussed with the other authors (AP, VW, AS, and NN) during a consultation. We particularly adhered to the Joanna Briggs Institute (JBI) protocol for scoping reviews [12].

**Fig 1:**
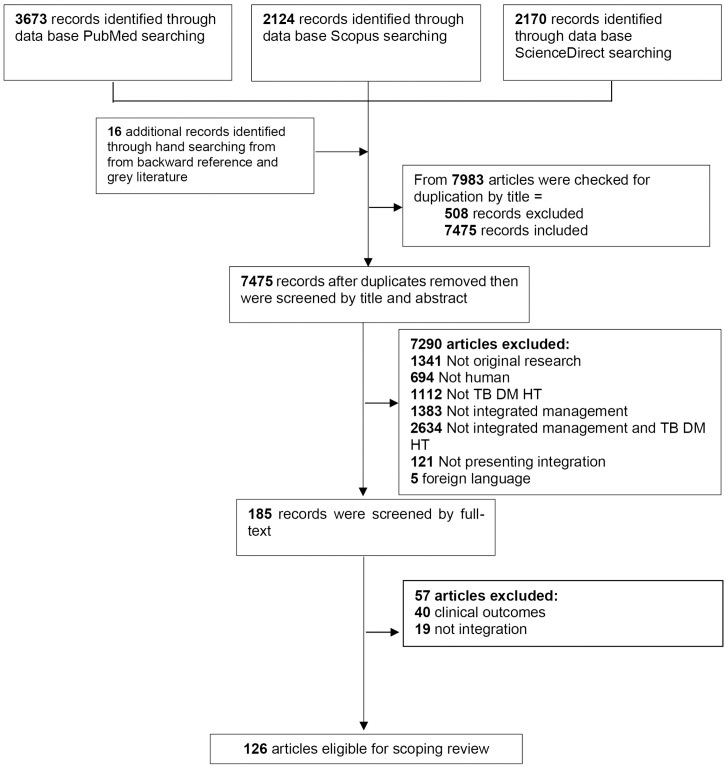
Flowchart of selection studies.

The flow of studies from initial identification to final inclusion is presented in Fig 1. The primary search of this review, conducted across PubMed, Scopus, and ScienceDirect, yielded 7983 studies, which were checked for duplicates by title. After removing duplicates (n = 508), 7475 studies remained and were screened by title and abstract. Of these, 7290 articles were excluded, leaving 185 articles for full-text screening. During full-text screening, 57 articles were excluded, 40 due to clinical outcomes and 19 for lack of integration in-between these diseases. Ultimately, 126 articles were deemed eligible for data extraction in this scoping review (Fig 1).

### Charting the data

Studies included in this review were classified based on the type of disease management (screening, treatment, others), barriers and facilitators related to the six WHO health system building blocks (policy and governance, financing, human resources, medicines and medical technologies, health systems delivery, and health information), and level of implementation in health care systems (community, primary, and referral care) [13]. Positive outcomes in the studies included are relevant to the integration and are defined as potentials. While any factors hinder the integration is defined as barriers (negative outcomes). Any efforts to tackle the challenges are included as strategies.

## Results

### Description of studies

Regarding the level of implementation, integration among TB, DM, and hypertension was notably conducted at primary care (n = 61, 46%), followed by community level (n = 49, 37%), then referral care (n = 24, 18%), with seven studies reporting multilevel integration [14–20]. Most studies (n = 48, 38%) originated from low- to middle-income countries. The remaining studies were relatively evenly distributed across high-income (n = 27, 21%), low and middle-income (n = 26, 20%), and upper-middle-income countries (n = 22, 17%). The concept of integration consisted of health promotion (n = 6, 7%), screening (n = 61, 56%), diagnosis (n = 11, 10%), and treatment (n = 32, 29%). The list of all included papers was provided in the S1 Table.

**Fig 2:**
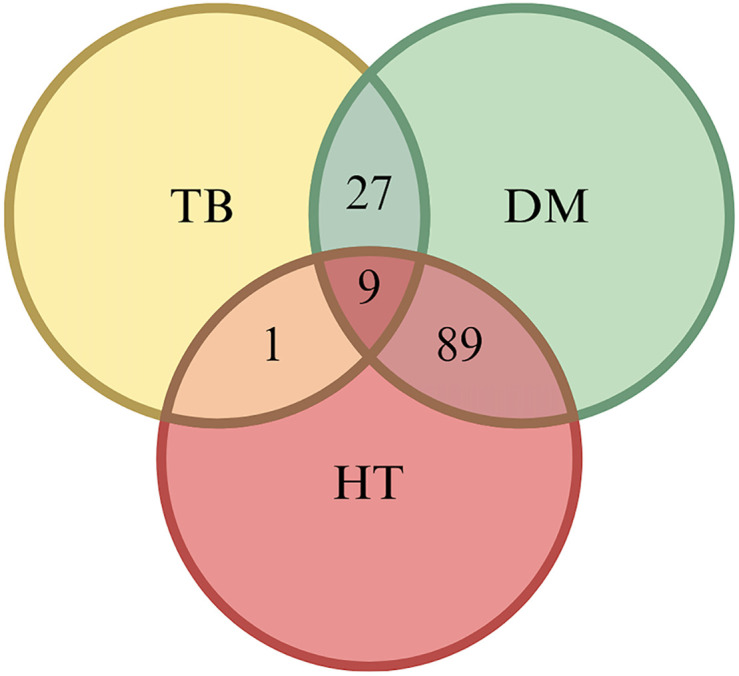
Number of selected studies based on the type of diseases in the integrated case management.

Only nine studies (7%) cover the integration of three diseases (TB, DM, and hypertension) (Fig 2). Most studies reveal the integrated management of two diseases (TB and DM, TB and hypertension, or DM and hypertension). The integrated management also exists with other chronic diseases such as HIV, renal diseases, and mental health. Among the nine studies of integrated management of TB, DM, and hypertension [21–29], five studies showed the integration at the community level [24–28], focusing on screening, and one study was conducted at the primary healthcare level (n = 1, 12.5%) [29], and three studies were conducted at the referral healthcare level (n = 3, 37.5%) focus on integrating NCDs in TB care [21–23].

The integration between two disease control programs/health services occurred mainly between DM-hypertension (n = 89, 71%), while the integration between infectious and non-communicable disease management (i.e., TB-DM and TB-hypertension) is less common (n = 28, 22%). The integration between DM-hypertension is mainly done at the primary health care level (n = 61, 46%), focusing on screening and treatment. Meanwhile, there are 36 studies on community-based and fifteen studies at the referral level. For TB-DM integration, there are ten studies in the community, eighteen studies in primary health care, and nine studies at the referral level for TB-DM integration.

The literature comprised of observational studies (cross-sectional/cohort; n = 40) and qualitative studies (n = 30) as the largest groups, followed by RCT/experimental studies (n = 23), prevention program evaluations (n = 14), mixed-methods studies (n = 11), and other designs (n = 8). Across the 126 included studies, 123 studies (97.6%) reported at least one potential (positive outcomes). Conversely, 21/126 studies (16.7%) did not report any barriers (negative outcomes), and 3/126 studies (2.4%) did not report any potentials. This suggests that positive outcomes were almost universally captured across the literature, whereas a meaningful minority of studies focused on outcomes without documenting negative effects. By study design, studies without any barriers (n = 21; 16.7% of all studies) occurred most often in observational designs (6/40; 15.0%), followed by prevention program evaluations (5/14; 35.7%) and RCT/experimental studies (4/23; 17.4%). Smaller numbers were observed in qualitative studies (3/30; 10.0%) and other designs (3/8; 37.5%). In terms of the presence of barriers, this corresponds to 34/40 observational (85.0%), 27/30 qualitative (90.0%), 19/23 RCT/experimental (82.6%), 9/14 prevention program evaluations (64.3%), and 5/8 other designs (62.5%) reporting at least one barrier. For positive outcomes, only three studies overall did not report any potentials (3/126; 2.4%), comprising 2 observational studies and 1 qualitative study. Accordingly, 38/40 observational studies (95.0%) and 29/30 qualitative studies (96.7%) reported at least one potential. This implies that all RCT/experimental (23/23), prevention program evaluations (14/14), mixed-methods studies (11/11), and other designs (8/8) reported at least one potential.

The literature analyzed the settings of integration, consisting of urban (n = 49; 38.8%), rural (n = 38; 30.2%), and mixed urban and rural settings (n = 39; 31%). Therefore, in total, there are 34 studies conducted in rural settings and 33 studies conducted in urban settings that reported barriers. Among these studies, 34 studies in rural areas reported barriers, which mainly include limited availability of diagnostic tools and digital resources, restricted access to specialized care, and less structured health systems. Meanwhile, 33 studies in urban areas reported barriers, primarily related to high patient volumes, despite having better resources and more organized healthcare systems that support integration efforts [30–34]. In contrast, rural settings are frequently constrained by limited healthcare infrastructure, workforce shortages, and geographic inaccessibility, although they benefit from stronger community engagement and participation. Studies conducted in urban-rural settings generally reflect a combination of these characteristics, balancing broader service coverage with persistent disparities in resource availability and service delivery [35–41].

A total of 16 studies explored the economic evaluation of strategies involving shared infrastructure, task-shifting, and human resource optimization [15,25,33,41–51]. Among these, all studies incorporated elements of human resource optimization (n = 16, 100%), reflecting a consistent focus on improving workforce efficiency and service delivery. Within this group, task-shifting and service integration, such as group medical visits, redistribution of clinical responsibilities, and integrated management of multiple conditions, were described in nine studies (n = 9, 56%). These approaches demonstrated substantial efficiency gains, including reported cost savings for integrated visits and group-based care models. Meanwhile, shared infrastructure or integrated service platforms (combining HIV, TB, hypertension, and diabetes services within single systems) were identified in seven studies (n = 7, 44%), often associated with reduced per-patient costs and improved cost-effectiveness outcomes.

Specifically, twelve studies (n = 12, 75%) demonstrated that the interventions were cost-effective based on established willingness-to-pay thresholds [25,45–48,50–52]. Additionally, three studies (n = 3, 19%) explicitly reported reductions in catastrophic or direct household costs, including one study showing a 20% reduction in tuberculosis-related household expenses through cash transfers and another highlighting that only 5% of patients experienced catastrophic costs under integrated care approaches. Furthermore, two studies (n = 2, 13%) addressed loss to follow-up or continuity-related economic benefits indirectly through improved adherence mechanisms such as multi-month dispensing and peer-support [15,25,49] models. Overall, these findings indicate that while human resource optimization, particularly task-shifting, dominates the evidence base, shared infrastructure and integrated care models consistently contribute to improved economic efficiency, reduced patient financial burden, and better continuity of care.

### Potentials

Integrated management of TB, DM, and hypertension has demonstrated several potential benefits, including enhanced access to care, improved continuity and retention in care, early detection and management of diseases and comorbidities, and improved cost-effectiveness.

#### Improved case detection and access to care.

Numerous studies have demonstrated that integrated disease management can enhance access to healthcare, particularly through expanded screening coverage that enables earlier diagnosis and timely treatment [22,52–57]. However, evidence suggests variations in effectiveness based on resource availability. In low-resource settings and rural areas, integration serves as a critical entry point for patients who otherwise lack specialized NCD care, yet its success is frequently constrained by external structural barriers [38,58]. For instance, while integrated screening improves detection in rural Ethiopia and Tanzania, the continuity of care is often broken by external factors such as the transportation costs, high out-of-pocket costs, and recurring medication stock-outs that integration alone cannot resolve [15,33,38,58,59]. Conversely, urban and higher-income settings, such as those in Central Vietnam or Indonesia, demonstrate more effective follow-up and disease control because the foundational infrastructure (stable supply chains and digital health information systems) is already in place to support the newly integrated protocols [36,61]. Early detection facilitates more effective disease management. Improved follow-up care has been identified as a key component of the care continuum, supporting treatment adherence, monitoring progress, and enabling timely adjustments, all of which contribute to improved health outcomes [60–65].

Access to healthcare has also seen notable improvements. Greater accessibility enables more patients to receive appropriate and timely medical attention, which is especially important for managing both acute and chronic conditions [22,24,52–57,66,67]. The increase in diagnosed patients reflects the success of these integrated efforts. With more individuals identified, healthcare systems are better positioned to allocate resources efficiently and implement targeted interventions [24,56,64].

Moreover, the integration of technology into screening and disease management programs has further enhanced their effectiveness. Digital tools improve data collection, streamline clinical processes, and support patient engagement, all of which are essential components of modern healthcare delivery [18,56,62,68–72]. Evidence suggests that these digital interventions currently achieve higher impact in urban centers and middle-income countries due to superior telecommunications infrastructure and higher digital literacy among both providers and patients [56,64,73]. In these settings, high digital penetration allows for seamless EMR systems and patient-generated health data (PGHD) to bridge the gap between home and clinic [56,64,70,73]. Conversely, in rural and low-income countries, the digital approaches remained a significant barrier, while technology offers the theoretical potential to bypass geographic isolation, its effectiveness is often stifled by frequent power outages, inconsistent internet connectivity, and a lack of technical support for frontline health workers [69,72–74]. Integrated disease management improves healthcare access through expanded screening coverage, improved follow-up mechanisms, and more strategic resource allocation [18,56,62,69,71,72,74]. However, to sustain and amplify these benefits, it is essential to invest in supporting infrastructure, including capacity building and robust health information systems, particularly for the integrated management of multimorbidity.

#### Early detection and management of diseases and comorbidities.

Early management of diseases and comorbidities has shown promising results in improving the detection and management of patients with TB, DM, and hypertension [21,22,31,52–57,74–76]. With early management, healthcare providers can offer appropriate treatments and support, prevent further deterioration, and improve health outcomes [21,52,54,56,77–80]. Early referral also ensures patients receive the right care at the right time, benefiting both them and their families by managing symptoms better and improving quality of life [24,56,64,78–80]. Data from the analyzed studies indicate that these systems work most effectively in urban and higher-income settings. In these areas, referral facilities are better prepared with specialized staff and functional diagnostic tests, such as HbA1c and creatinine testing. In these urban centers, early detection leads to immediate clinical action [43,75]. In contrast, in low-income rural settings (such as parts of Ethiopia and Cambodia) early management often meets barriers due to equipment inertia and the lack of secondary-level laboratory readiness [37,38,40,59]. Consequently, although urban health systems often face challenges related to high patient volumes and fragmented continuity of care in managing two or three diseases in one site despite improved screening, rural health systems frequently struggle to translate early diagnosis into effective treatment, as essential referral services remain physically distant and financially inaccessible for many patients [29,35–40,58,59].

The integration of healthcare services introduces a comprehensive and patient-centered approach to managing DM and TB treatment synergistically [14,32,52] and providing more personalized care [52]. Although research on the integration of TB, DM, and hypertension is still limited, existing evidence suggests that integrated care can lead to better health outcomes and improved quality of life for patients [14,32,52,61]. There is no study showing that integration can reduce the adverse effects of multimorbidity.

#### Improve the efficiency of health services.

Some studies revealed that the integration of TB and DM management is cost-effective to reduce financial strain on healthcare systems and to improve patient compliance with treatment [9,15,44,45,47]. The integration of TB and DM services also has the potential to optimize medication regimens and minimize unnecessary clinic visits, which can enhance the efficiency of care delivery [19,44,81–83]. This approach not only alleviates the workload of health staff [19,30,84–86]. Tu et al. (2020) explain that early detection and effective management of NCDs reduce their impact on individuals and communities, promoting healthier lives and lowering healthcare costs [89].

#### Improve continuity of care and retention in the program.

Enhancing continuity of care and patient retention within healthcare programs is essential for achieving long-term health outcomes [36,58,74,87–89]. A growing body of literature underscores the effectiveness of structured follow-up protocols in maintaining patient engagement and adherence to treatment plans[36,56,58,62,74,83,90]. The use of digital technologies, such as automated reminders and telehealth services, has been shown to reduce missed appointments and improve the overall consistency of care delivery [56,62,76,80,91–94]. Personalized follow-up strategies further contribute to these outcomes [49,60,77,83,90]. Tailoring follow-up care to individual patient needs—such as by considering cultural factors, health literacy, and patient preferences—has been associated with increased adherence rates and better clinical results [49,60,77,83,90]. Central to these efforts is the use of comprehensive data tracking and integrated healthcare systems, which allow for seamless communication among multidisciplinary care teams and support informed decision-making [18,56,62,74,95–97].

Effective communication between healthcare providers and patients, along with active involvement of patients’ support systems, is another cornerstone of integrated care. Empowering patients through education and support enhances their capacity for self-management, which in turn contributes to improved functional outcomes and overall well-being [58,60,77,83,90,98,99]. Tu et al. (2020) highlights the importance of patient motivation, education, and consistent provider support in fostering adherence to both medication and healthy lifestyle behaviors [89]. Retention in healthcare programs also hinges on relational and systemic factors. Personalized communication strategies and consistent, proactive follow-up are essential for sustaining patient engagement over time [58,60,77,83,90]. Additionally, the integration of medical records ensures that all healthcare professionals involved in a patient’s care have access to a complete and up-to-date health history, thus facilitating better coordination and continuity [18,56,95–97,100].

Patient-provider interactions also play a pivotal role in influencing perceptions of care quality. Studies by Mohr et al. (2019) and Benzer et al. (2019) suggest that positive interpersonal experiences within the healthcare system can significantly enhance patient satisfaction and trust, further supporting retention and adherence [91,92]. Finally, addressing systemic barriers, such as the financial cost of preventive screenings, is crucial for promoting equitable access to care [15,25,49,53,58,101]. Basir et al. (2019) and Rosenberg et al. (2020) emphasize that reducing these barriers and fostering a culture of proactive health management can improve participation in preventive health measures, ultimately leading to better public health outcomes [25,53].

#### Improve cost effectiveness.

The findings of this review indicate that integrated care models are generally economically favorable across diverse settings [25,42,43,45–48,50,51]. Most included studies demonstrated cost-effectiveness, with incremental cost-effectiveness ratios (ICERs) ranging from less than $50 per disability-adjusted life year (DALY) averted to approximately $23,700 per quality-adjusted life year (QALY), suggesting that these interventions fall within commonly accepted willingness-to-pay thresholds [45–48,50,51]. These results highlight the potential of integration strategies to deliver meaningful health gains while maintaining efficient resource use [25,42,43,45,46,48,50,51].

A key driver of these economic benefits is the ability of integrated models to leverage existing existing resources, particularly through shared infrastructure, task-shifting and human resource optimization, including reduced catastrophic costs and loss to follow-up [25,42–44,48,50,51,85,86]. In the South African and Eswatini studies, the clinics, supply chains, and staff were already funded for HIV care [42,43]. Adding hypertension or DM screening allowed the system to achieve economies of scope, where the total cost of providing two services together is lower than the cost of providing them separately [42,43,50,51]. By utilizing the same physical space and administrative staff, health systems in Eswatini reduced the cost of a chronic care visit from $10.85 to $6.53, a nearly 40% reduction in service delivery costs [42].

Integrated models frequently rely on task-shifting, where lower-level healthcare workers take on routine monitoring tasks [30,84–86]. As seen in one study in Kenya, using group medical visits allows one clinician to see 10–15 patients at once rather than 10–15 separate appointments [48,73]. This drastically reduces the personnel cost per patient, which typically accounts for 50–60% of a clinic’s budget [43]. Although the time allocated per patient may be reduced, the depth and meaningfulness of patient engagement often improve through shared experiences and peer support, ultimately enhancing the overall efficiency of care delivery relative to the resources invested in personnel [25,48,73].

A key systemic benefit highlighted in the Manitoba and China studies is the substantial cost-saving achieved through the prevention of catastrophic complications [46,47]. From a health system perspective, the cost of a point-of-care (POC), such as glucose or blood pressure test is negligible (often under $2.00) compared to the catastrophic cost of treating a stroke, heart attack, or end-stage renal failure [42,46]. In remote Indigenous communities, for example, the system saves millions of dollars in medical evacuation and dialysis costs for every patient whose kidney disease is managed early through integrated screening [46,70,102]. This shifts the financial burden from expensive hospital care to affordable primary care.

In fragmented systems, patients often drop-out of the care pathway when they are referred from one clinic to another (e.g., from a TB clinic to a separate DM center) [36,58,64,103,104]. This missed opportunity of diagnosis and treatment is a high burden for the health system [58,64,103,104]. Integrated models, like the mobile units in Peru, achieved linkage rates as high as 93% for TB and 81% for hypertension [26]. By closing the gap between screening and treatment in a single visit, the system ensures that the initial investment in diagnosis results in a treated patient, maximizing the return on diagnostic investment [24,26,45,51].

#### Barriers and key strategies.

**Fig 3:**
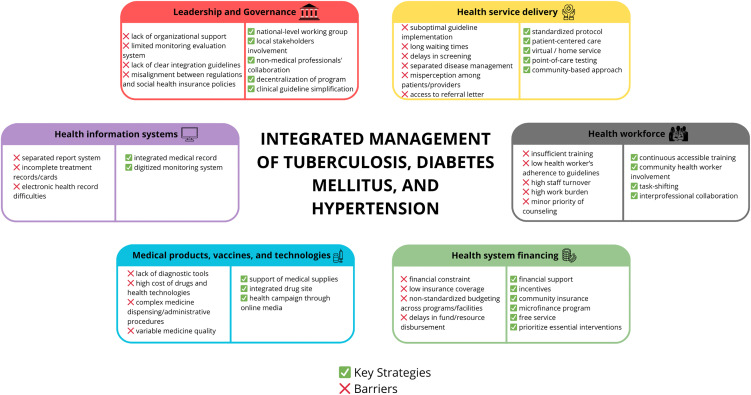
Summary of the barriers and key strategies of TB, DM, and hypertension integrated management.

We describe barriers and key strategies based on the six building blocks of Health Systems by WHO. Details of barriers and key strategies are presented in S2 Table. Summary of the barriers and key strategies of the three diseases integration are provided in Fig 3.

### Leadership and governance

#### Barriers.

Integration of disease management needed a roadmap to be a crucial step in making a strategic plan for a health program that is expected to ensure the effectiveness of an implementation. It can be delayed for several reasons: external interference and non-specific guidelines [19,23,38,105–109]. Inadequate guidelines remain a significant challenge in healthcare systems, particularly in LMIC. Research highlights that the absence of well-defined, evidence-based, and locally tailored guidelines hinders effective healthcare delivery [19,23,38,105–109]. Previous studies emphasize the need for robust frameworks to guide clinical practices [19,23,38,105–109]. Without such frameworks, healthcare providers face difficulties in standardizing care, leading to inconsistent outcomes and inefficiencies [19,23,38,105,107,108].

The problem is further exacerbated by ambiguous governmental strategies, alongside insufficient support and leadership from the pertinent organizations [19,23,105–111]. Some studies underscore the importance of strong governmental commitment and leadership in implementing healthcare initiatives [19,23,105–107,109–111]. The lack of strategic direction often results in fragmented efforts, where policies fail to align with on-the-ground realities [19,23,38,105–109]. This misalignment varies significantly across economic contexts in LICs like Ethiopia and Malawi, leadership gaps often result in a total lack of essential screening and diagnostic equipment (e.g., glucose sticks) and medications [17,23,40,59,104–106]. Conversely, in middle income countries such as India, Indonesia, and Vietnam, the challenge shifts from resource scarcity to a lack of coordination between robust public and private sectors, leading to doctor shopping and data fragmentation [36,38,112,107,113]. These gaps lower the effectiveness of interventions and erodes trust among stakeholders [19,23,36,38,105–111].

Another critical issue is the absence of systems to monitor and evaluate the integration of healthcare services [18,23,56,71,96,97,105]. Monitoring frameworks are essential to assess progress, identify gaps, and ensure accountability [18,23,56,71,96,97,105]. Several studies highlight that without proper evaluation mechanisms, it becomes challenging to measure the success or shortcomings of integration efforts [18,23,56,71,96,97,105]. This lack of oversight undermines efforts to improve healthcare delivery and limits opportunities for continuous improvement [18,23,71,96,97,105]. Better data collection is also crucial for informed decision-making [18,56,71,95–97].

Lack of a content-specific guideline; for example, DM-HT-specific plans rather than NCDs in general [19,23,38,69,105,106,108,109]. This is important for healthcare managers to understand their leadership roles and commitments and to understand the big roadmap instead of focusing on their mechanisms [19,23,38,69,105,106,108,109]. In addition, a country’s health goals should be prioritized by local evidence from in-country research and not influenced by external funding organizations [38,105–107,109,111,114].

#### Key strategies.

In terms of policy, integrated disease management needs collaboration with the health authorities, supporting organizations or communities, having specific-explicit guidelines [19,20,23,38,40,57,69,105,106,108,109], and decentralization [28,30,32,72,84–86,93,115,116]. Partnership will result in a continued activity, improve patient health indicators and increase participation [41,61,73,110,111,116–121]. Financial constraints were less likely to be found in supported programs [25,42–44,48,50,51]. Engagement with providers and communities will strengthen the interaction with the community, building awareness and trust [19,30,33,41,58,60,70,83,118,120]. Community participation in the planning and implementation of health centers is suggested to improve the quality of care and to create a transparent accountable system [27,28,30,33,41,73,120]. Lastly, embedding integrated disease management into a national disease control strategy will make these programs more scalable and sustainable [9,18,38,69,105,109,122–134].

The guidelines are implemented as diagnostic and management guidance directly [18,21,23,57,69,105,106,108,109,122–125,133–136] or in the form of a digital application [18,56,62,137,92–95,97,115,138]. Integration of programs into pre-existing clinics is favored to reduce existing limitations, such as a lack of human resources or infrastructure [17,32,41,50,51,61,71,72,84–86,110,116,118,139]. Further, combined with technology support, decentralized care also addresses the critical shortage of health service providers [18,28,30,32,56,72,84–86,92,93,115,116]. While both settings benefit, digital interventions yield the most transformative gains in rural areas by bypassing geographic barriers and lack of diagnostic infrastructure [32,33,66,80,92,93,115,116,138], given digital support is adequate. Conversely, in urban settings, digital support primarily addresses the complexities in data recording and reporting as well as fragmented care across multiple providers, ensuring longitudinal continuity [18,36,56,62,75,92,95,97,107]. The key to successful decentralization is mentoring of the health workers [28,30,31,33,84–86,93,115,119,140,141]. Efforts also include collaborations between academic institutions, healthcare facilities, and community-based programs to enhance diabetes and hypertension management [31,48,73,119–121,140–144].

### Health service delivery

#### Barriers.

Several factors can hinder effective healthcare delivery and management. One challenge involves expensive partnerships with the private sector, which can strain resources and potentially prioritize profit over patient care [16,110,146]. Many individuals face a lack of access and financial support when seeking referral care, creating barriers to necessary medical services [15,17,23,29,36–39,49,58,64,69,90,101,103,105,110]. Poor clinic health systems further exacerbate these issues, potentially leading to inadequate care and management [17,23,34,37,38,40,41,59,104,105,110].

Inefficient processes also contribute to these challenges, as healthcare interventions can be time-consuming for both providers and patients [14,23,29,34–37,39,41,58,64,90,145]. A limited awareness of collaborative frameworks and separated disease management approaches can hinder coordinated care efforts [17,19,23,38,81,103,105–110,118]. The absence of early detection and management systems can result in delayed interventions and poorer health outcomes [19,21,23,40,52,54,59,103,104,135,146–149]. Barriers like long waiting time were also faced in the integration of diseases [14,23,29,32,34–37,39,41,58,74,90].

Furthermore, poor recording and reporting systems can compromise data accuracy and hinder effective monitoring and evaluation [18,23,56,71,81,95–97,105,107]. Suboptimal guideline implementation can lead to inconsistencies in care delivery and missed opportunities for evidence-based practice [19,23,38,57,81,105,106,108,109,135]. A lack of emphasis on physical or dietary interventions may limit the scope of treatment and preventive care [32,60,77,83,137,90,142]. The loss of referral letters, limited DM services, and long waiting times further impede access to timely and appropriate care [23,29,36,37,39,40,58,59,64,90,103–105]. Inconsistent communication, such as reminders not being delivered to intended patients and retention by specialists, can disrupt continuity of care [36,56,58,64,74,88,89,103].

#### Key strategies.

Improving healthcare delivery for patients with chronic and infectious diseases requires a multifaceted approach that includes referral systems, counseling, and service integration. Strengthening referral mechanisms to higher-level health facilities and enabling two-way referral systems ensures continuity of care and proper case management [24,26,30,35,36,52–57,58,64,69,72,74,77,137,103,104,115,119,150–152]. At the community level, counseling and health education are essential for improving patient awareness, engagement, and treatment adherence]. Integrating services—such as for TB, diabetes, and NCDs—creates more efficient care pathways and reduces fragmentation [9,17,21–24,26,31,32,36,41,50–54,112,61,67–69,71–74,80,81,84–86,104,105,110,115,116,118,152–155]. Supporting this, chronic disease outreach programs, post-discharge community care, and home visits extend care access beyond traditional clinical settings [24,26–28,32,46,48,66,67,70,84,94,102,116,121,156,157].

Advancing care quality also involves implementing standardized protocols, algorithm-based treatments, and clinical decision support systems to guide evidence-based practices [18,20,21,23,56,57,112,69,76,92,109,122–125,133–136,150,153,158]. Tools such as simple screening forms, mobile health applications for reminders, and point-of-care testing improve efficiency and early diagnosis [18,21,24,26,27,45–47,54–57,62,66,67,76,80,92–95,97,115,135,138,142,149,150,159]. Moreover, patient-centered care—including individualized medication plans, tailored education, and peer-group-based interventions—ensures that care is responsive to individual needs and improves self-efficacy [14,25,38,48,49,60,68,70,73,77,78,82,83,137,90,92,119,121,142,157]. Digital health innovations, like virtual consultations, mobile phone apps for tracking lifestyle and self-measurements, and home-based or mobile screening clinics, enhance accessibility and promote patient self-management [18,24,26,27,32,33,46,56,62,66,67,76,80,91–95,97,102,115,116,121,138,142].

Furthermore, strategies that target early detection and underserved populations—such as active case finding, targeted and home-based screening, and developing tailored screening models—are vital to reducing diagnostic delays and addressing health disparities [21,22,24,26–28,45–47,52–55,66,67,70,92,116,135,147–149,159,160]. Special screening efforts like TB screening in high-risk DM patients, mental health screening for HIV/NCD patients, and group medical visits with community health workers add further depth to patient-centered care [25,28,47,48,73,92,121,141,161]. Ensuring after-hours clinic availability, promoting community-based approaches, and coordinating with specialists round out a comprehensive framework that prioritizes continuity, personalization, and accessibility in healthcare services [24–28,30,33,36,41,58,60,64,66,67,70,74,84,88–90,103,116,119–121,155].

### Health workforce

#### Barriers.

Healthcare service delivery is often challenged by significant human resource constraints. A major issue is chronic understaffing, which is exacerbated by high staff turnover, frequent absenteeism, and a heavy workload borne by the remaining personnel [19,23,33–35,37–41,55,162,59,81,83,84,93,105,108,110,154]. These conditions are further strained by short training durations [19,23,33,34,38–40,57,59,84,93,104,105,108] and limited opportunities for skill development, leading to competency gaps among healthcare workers [19,21,23,33,34,38–40,57–59,162,71,84,93,104,105,108]. Inadequate training not only undermines care quality but also contributes to poor adherence to clinical protocols [19,23,33,34,38,39,57,59,84,86,104,105,108]. As a result, service efficiency suffers, increasing the risk of burnout and compromising patient outcomes [19,23,33–35,39–41,162,59,84,93,105,108,110].

In addition to structural limitations, behavioral and organizational factors also affect healthcare performance. The lack of interprofessional collaboration [18,38,70,74–76,78,105–109,118,145,150,153,158] reduces the effectiveness of integrated care, while certain provider attitudes—such as overconfidence [16,86,146], unwillingness to engage [16,17,19,23,105,107,110,118], and the failure to prioritize counseling [19,60,77,83,84,90]—can hinder patient-centered care. The persistent risk of infection, especially in high-burden settings, further compounds these challenges [19,21,23,81,103,107,135]. Together, these issues highlight the need for comprehensive workforce strategies that include ongoing training, better staffing models, and systems that support collaboration and provider accountability [18,19,23,30,31,33,34,38,57,84–86,105,108,110].

The integration of management by health workers presents a significant challenge to implementing effective health programs caused by human resource limitations [19,23,30,31,33,34,38,40,57,59,84,105,108,110]. This has led to the concept of empowering community health workers (CHWs) to bridge this gap [27,28,30,33,73,84–86,119,121,140,141] especially in LMICs. To effectively utilize CHWs, several factors must be considered [28,30,33,73,84–86,119,121,141].

Engagement in care is influenced by personal motivation, patient-provider relationships, and social support. Key facilitators include personal initiative, education, positive provider-patient interactions, and support from family and community. Integrated care services and social support are essential for enhancing patient engagement and adherence in LMICs [25,30,33,58,60,61,73,83,90,118].

#### Key strategies.

Strengthening human resources through targeted capacity-building interventions is essential for improving healthcare delivery. One of the most frequently implemented strategies is the training of both health workers and non-health workers, which plays a critical role in enhancing knowledge, skills, and service quality [19,21,23,28,30,31,33,34,38–41,55,57,162,59,83–86,93,104,105,108,110,119,140,141]. Training methods have expanded to include continuous and accessible formats, such as web-based learning [18,56,96], and are often tailored to be context-specific and user-friendly, particularly for community health workers (CHWs) [28,30,33,85,86]. These efforts are further reinforced by formal linkages to professional associations, which help maintain standards and peer support networks [121,144].

In addition to training, several complementary strategies support workforce optimization. Task-shifting—delegating certain clinical tasks to trained lower-level workers—has proven effective in addressing workload challenges and improving service access [18,27,28,30,33,84–86,119,121,141]. Task shifting from healthcare workers to community workers requires strict supervision, including community visits and routine evaluations. CHWs should be selected by local leaders and equipped with adequate knowledge and resources, supported through remuneration. Facilities may include dedicated buildings for CHWs, phones, and transportation allowances [27,28,30,33,73,85,86,119,121]. Additionally, specialty-trained CHWs can enhance patient trust and confidence [28,30,33,119,121,140,141]. Non-physicians can also play a role in integrated disease management by conducting motivational interviewing and monitoring patients with comorbidity conditions, which has been shown to improve treatment outcomes [27,28,30,33,73,83–86,121,140,141].

In some settings, hiring additional temporary staff has helped alleviate workforce shortages [34,84]. Moreover, interprofessional collaboration—involving dieticians, nurses, pharmacists, and counselors—enhances integrated care delivery and supports a more holistic approach to patient management [18,38,70,74–76,78,105–109,118,143–145,150,153,158]. Community health workers play a vital role in ongoing patient support, particularly when empowered with proper guidance, training, and integration into care teams [27,28,30,33,73,84–86,119,121,140,141]. Together, these strategies create a more resilient, adaptive, and patient-centered health workforce.

### Health information systems

#### Barriers.

Challenges in health information systems continue to hinder efficient service delivery and patient management. One common issue is the incomplete documentation of treatment cards, which compromises the accuracy of patient records and continuity of care [57,82,105]. In addition, many healthcare workers face difficulties operating electronic medical records due to a lack of training or system complexity [56,62,93,95–97,115]. Even when electronic medical records are used, their functional limitations often restrict their ability to support comprehensive data management and care coordination [95–97,115]. In some cases, the absence of individual patient records further complicates the ability to track treatment progress and outcomes [23,71,96,105].

Fragmentation within health information systems also contributes to inefficiencies. The use of separated reporting systems creates duplication of work and reduces the integration of critical data across services [23,29,81,82,95–97,105,107]. This is compounded by poor data management practices, which lead to inconsistencies and reduce the reliability of health information for decision-making [18,23,71,81,82,95–97,105,107,111]. Furthermore, difficulties in data retrieval and cross-verification limit the ability to validate and analyze data accurately [82,95–97]. Addressing these systemic issues is essential to ensure accurate documentation, streamline workflows, and support evidence-based care planning [18,56,71,93,95–97,115].

#### Key strategies.

The use of digital tools and electronic medical records (EMRs) is increasingly recognized as a critical component in enhancing healthcare service delivery and data management. Electronic medical records support accurate documentation and streamlined access to patient information, facilitating better clinical decision-making and continuity of care [18,56,62,71,92–97,115,138]. Alongside electronic medical records, traditional treatment cards remain in use as supplementary tools for tracking patient care, especially in settings where full digitization is not yet feasible [56,57,76,82,105,135]. Some systems also employ parallel registration methods that integrate a patient’s medical history to improve coordination between services [52,56,95,96]. In addition, digitized monitoring systems enable real-time tracking of treatment progress and patient outcomes, which strengthens follow-up and program evaluation [18,36,56,62,71,92–97,115].

To enhance the functionality of these systems, healthcare providers are adopting integrated technology platforms that assess patient risk levels and generate tailored care recommendations [18,56,62,92–95,97,115]. These platforms often combine multiple functions, such as incorporating medical guidelines, health insurance details, and medicine availability into a single, integrated medical record [18,56,95–97,115]. Furthermore, electronic applications designed to support guideline-based assessments and clinical management are being implemented to improve adherence to standards of care and reduce clinical errors [18,56,62,92,93,95,97,115]. These innovations contribute to a more efficient, coordinated, and patient-centered healthcare system, especially when aligned with supportive infrastructure and training [18,56,62,71,92–97,115,138].

### Medical products, vaccines, and technologies

#### Barriers.

A key barrier to effective healthcare service delivery is the shortage of essential medications and diagnostic equipment. Many facilities report frequent stockouts of both medicines and tools needed to properly evaluate and manage patients [14,16,17,19,21,23,33–35,37–41,57,162,59,83,84,93,105,108,110,135,154]. In several cases, diagnostic equipment is either unavailable or insufficient, limiting the ability to conduct timely and accurate assessments [20,23,37,38,40,59,105]. These resource gaps undermine clinical decision-making and compromise patient care, particularly in high-burden or under-resourced settings [17,19,23,33,34,37,38,40,59,105,108].

Cost-related issues further exacerbate access challenges. The high price of drugs and expensive medical technologies makes it difficult for both healthcare systems and patients to afford consistent treatment and diagnostic services [15–17,25,37,39,49,58,101,105,110]. Additionally, inefficient procurement systems, including the lack of standardized procedures for medicine requests, contribute to recurring shortages and delays [23,81,105,108,109,111]. In certain settings, the availability of non-standard or low-quality medications introduces an additional layer of concern, potentially impacting treatment efficacy and patient safety [16]. Addressing these supply chain and cost barriers is crucial to ensuring reliable, high-quality care across all levels of healthcare [16,23,38,59,81,105,108–111].

#### Key strategies.

To strengthen healthcare delivery, ensuring the availability of essential medical equipment and medications is a critical priority. Efforts to provide the necessary medical tools and diagnostic equipment can significantly improve service readiness and patient care quality [20,23,38,40,57,59,84,105]. Similarly, ensuring consistent access to required medications, particularly for chronic and infectious diseases, helps maintain treatment adherence and reduces the risk of complications [14,16,17,38,39,49,57,59,83,105,108,110]. Establishing integrated, centralized drug collection points has also proven effective in improving medicine distribution efficiency and access, especially in underserved areas [17,32,85,87,116,149].

### Health system financing

#### Barriers.

Financial limitations remain a significant barrier to effective healthcare delivery. Health systems often face budgetary constraints, including unstandardized budget allocations across facilities and delays in the distribution of allocated funds, which can disrupt service planning and delivery [23,33,34,37–41,49,58,59,162,83,101,105,108,110,111]. These systemic issues limit the capacity to maintain essential services, hire adequate staff, and procure necessary supplies [23,34,38,40,59,105,108,110,111]. Inconsistent financial planning across healthcare institutions further contributes to inequities in service provision and weakens the overall efficiency of health programs [101,111].

On the patient side, financial barriers significantly affect access to care and adherence to treatment. Many patients struggle with out-of-pocket costs, particularly in the absence of adequate insurance coverage [15–17,25,26,38,39,49,57,58,60,83,101,110,149]. These financial difficulties often lead to reduced medication adherence, delayed treatment, or the selective provision of medications based on a patient’s ability to pay [16,39,49,58,60,90,101,110]. Ultimately, these constraints not only affect individual health outcomes but also undermine broader public health goals. Addressing both systemic and patient-level financial barriers is essential to ensure equitable and sustainable healthcare access [15–17,25,38,39,49,58,101,110].

#### Key strategies.

Integrated screening is beneficial over a symptom-based approach due to the marginal costs for the integration being less compared to stand-alone screening programs [43,45,47,50,51]. Supports from the government was important to make the screening program integrated across diseases [38,104,109–111,122–134].

Funding opportunities in the existing health service delivery system can be a good opportunity to leverage resources for integration and to reduce patients’ out-of-pocket expenditure [25,42–44,48,50,51,110]. The government-supported insurance or microfinance or the implementation of community health insurance which is offered at a low cost might clarify the barrier cost [25,48,49,104]. This is further evidenced by the rising number of individuals seeking medical care in response to available incentives [24,25,27].

A study by Pastakia et al., showed focus on community-based care for hypertension and diabetes by targeting peer/microfinance groups, education, and treatment in the community by maintaining economic sustainability and incentives [75]. The results were substantial savings among participants and significantly impacted chronic disease care in low-resource settings [25,48,73]. Cost saving can be done by screening by targeting high-risk diabetic patients with specific factors like low body mass index, high fasting blood glucose, and low triglycerides for screening was found to be more cost-effective [47].

## Discussion

Integration of three diseases (TB, DM, and hypertension) is very promising, but nowadays integration is limited to two diseases. For example, only DM and hypertension or DM and TB. Combining two diseases into one integrated intervention is already challenging. The critical gaps include shortages of essential medicines, screening and diagnostic supplies, and equipment, as well as inadequate human resources. Furthermore, healthcare workers possess insufficient knowledge and skills required to effectively manage both diseases. Patient attendance remains low, compounded by limited awareness and insufficient understanding of the associated service delivery guidelines and operational frameworks [19,23,33,34,38,40,59,93,104,105,108]. Unfortunately, many global health donors give disease-specific funding such as NCDs or TB and rarely give funding that is integration-based [116], and the budget that the government gives is not adequate [23,34,59,105,108,110,111]. Integration has been proven to save costs [25,42,43,45–48,50,51]. Thus, health equity funds should be redesigned to ensure they provide adequate financial protection for patients living with NCDs [25,49,101]. However, the drivers of cost savings differ across settings. In urban areas, savings are often achieved through economies of scale, shared infrastructure, and higher patient throughput [42,43,50,51]. In rural areas, cost-effectiveness may arise from preventing expensive late-stage complications, reducing travel burden, and improving linkage to care through community-based delivery models [25,27,45–48,73]. Therefore, evaluations of integrated care should consider both provider costs and patient-incurred costs, especially in remote settings [15,25,42–44,46,48,49,101].

DM and hypertension integration is vast compared to other integrations. Understandably, as both DM and hypertension are NCDs, DM and hypertension integration is preferred over tuberculosis and NCDs integration due to the shared NCDs risk factors, higher population prevalence, and less stigma [63,76,137,87,163,164]. On the contrary, TB and hypertension case integration is the least due to their weaker direct connection. There are many guidelines for DM and hypertension [69,165,166] and TB and DM [122–125,133,167]. There are currently no guidelines that focus on TB and hypertension. Adding a third disease would require reworking indicators, patient records, reporting tools, and training health workers to record and act on more complex patient profiles [18,23,56,95–97,105]. Integration of three diseases, TB, DM, and hypertension, could find new cases of hypertension and diabetes among TB patients. This is one of the good forms of integration with NCDs and was considered feasible and acceptable [20–22,26,54,61,104,130,131].

In low-income countries, the implementation of integrated care models is often constrained by fundamental health system gaps. Several structural barriers persist, including a lack of screening and diagnostic equipment, as well as limited access to essential medicines and laboratory services, which are frequently not provided free of charge. In addition, weak health information systems are characterized by poor record-keeping, inadequate reporting mechanisms, and limited feedback and referral systems [17,23,34,37,38,40,57,59,104,105]. These challenges are compounded by a shortage of supporting agencies and implementation partners, as well as insufficient training among healthcare workers, which limits their capacity to effectively deliver integrated services [19,23,31,33,34,38,40,57,59,105,108,110].

Operational challenges are also more pronounced in these settings. Integration can lead to clinic flow delays and place additional strain on an already limited workforce, increasing workload without proportional resource expansion [19,21,23,33–35,40,59,105,108]. Furthermore, a lack of prioritization and insufficient allocation of resources to integrated programs can hinder their sustainability [19,23,33,34,40,59,105,108,110,114]. From the patient perspective, fear of screening or diagnosis may also arise when treatment options are unreliable or unavailable, reducing uptake of services and ultimately weakening the intended benefits of integration [16,17,23,38,39,49,52,58,72,105,107,110,149].

In contrast, while middle-income countries may have relatively stronger health system infrastructure, challenges tend to arise at the level of implementation efficiency. In these contexts, task-shifting may not always yield the desired outcomes. Health workers are often already overburdened, and the redistribution of tasks can exacerbate workload pressures rather than alleviate them [18,30,84–86,93,108,156]. Moreover, insufficient coordination, supervision, and role clarity can reduce the effectiveness of task-shifting approaches, leading to inefficiencies and potential declines in quality of care [19,23,30,33,38,41,85,86,105,107,108,110,158].

Key recommendations include strengthening health systems, promoting patient-centered care, and increasing funding for NCDs research. Proposed solutions involve task sharing, enhancing procurement practices, and utilizing older medications. Effective care delivery requires standardized guidelines, training, supervision, and robust data collection systems. The study stresses the importance of context-specific approaches, patient empowerment, and community-based interventions [18,23,30,32,33,38,57,69,73,85,86,93,105,109,110,114,168].

### Public health implications

Integrating TB, DM, and hypertension management requires a comprehensive and structured approach. Governments and health institutions need to develop policies that support the integration of these services [124–134,169,170]. Once policies are in place, the next step is to integrate TB, DM, and hypertension management at the healthcare facility level [20–23,61,104,124,125,130,131,152,170]. This means patients can receive comprehensive and coordinated care from a multidisciplinary healthcare team [18,75,78,118,143,144,150,153,171]. To support service integration, an integrated health information system needs to be developed [18,56,98–100,172]. This system can track and manage patient data for TB, DM, and hypertension, and facilitate communication between patients and healthcare staff [18,56,95–97,172]. Additionally, developing adequate human resources is crucial [19,23,30,33,34,38,84–86,105]. Healthcare staff need to be trained and developed to provide integrated management [18,19,23,30,31,33,34,38,57,84–86,105]. A multidisciplinary team needs to be developed to provide comprehensive care [18,77,80,120,144,145,151,154,171]. Adequate financing is also essential to support the integration of TB, DM, and hypertension management [25,42,43,50,51,110,173]. Innovative financing models need to be developed to ensure the sustainability of integrated services [25,42,43,48,50,51,173]. Integrating TB, DM, and hypertension management can lead to improved health outcomes, enhanced patient satisfaction, and more efficient healthcare delivery, then improving patient outcomes and reducing the disease burden in the community [9,21,22,50,51,63,72,76,107,153,154,170]. National implementation strategies should differentiate between urban and rural settings. Urban models may prioritize integrated facility-based chronic disease hubs supported by interoperable digital systems, where higher patient volumes and stronger referral capacity can improve efficiency and continuity of care [18,36,56,64,77,95,98,100]. In contrast, rural models may require task-sharing, mobile clinics, telehealth, and community health worker networks to overcome workforce shortages, transport barriers, and geographic inaccessibility [26–28,30,32,33,68,75,82,87,88,96,117,118]. Mobile and outreach-based integrated screening programs have also shown strong linkage-to-care potential for underserved populations, particularly where routine facility access is limited [24,26,27,68,69,118].

### Limitations and strengths

This is the first scoping review discussing the integration of TB, DM, and hypertension. This scoping review represents a significant undertaking in synthesizing the existing literature on the integration of TB, DM, and hypertension management. This scoping review can contribute to the field of public health, providing a comprehensive overview of the integration of TB, DM, and hypertension management. Our findings can inform policy development, practice improvement, and future research.

The limitations of this study relied primarily on qualitative studies when measuring the effects of integration. Experimental study designs, which focus on manipulating variables to establish causal relationships, provide stronger evidence but are often underrepresented in this review. While qualitative studies offer rich, contextual insights into behaviors and experiences, they cannot establish causality and are often limited in generalizability. This imbalance in research methodologies makes it difficult to draw clear and definitive conclusions about the impact of integration.

## Conclusions

This scoping review highlights the potentials, barriers, and key strategies of integrating TB, DM, and hypertension case management to improve case detection, continuity of care, patients satisfaction, and health system efficiency. Integrated approaches can reduce duplication of services and lower costs, particularly when supported by task-sharing, shared infrastructure, and strong referral systems. However, sustainability remains constrained by financing gaps, weak infrastructure, fragmented information systems, and workforce shortages. Importantly, implementation needs differ across contexts: urban settings may benefit from scale and stronger facility systems, whereas rural settings require decentralized and community-based approaches to overcome access barriers. Future research should prioritize long-term outcomes, implementation effectiveness, and comparative cost-effectiveness across diverse health system settings.

## Supporting information

S1 Table Description of selected studies. (DOCX)

S2 Table Barriers and key strategies of integrating TB, DM, and hypertension management. (DOCX)

S1 File Preferred reporting items for systematic reviews and meta-analyses extension for scoping reviews (PRISMA-ScR). (DOCX)
