# Supplementary material for: Potentials, barriers, and strategies for integrating tuberculosis, diabetes mellitus, and hypertension case management: A scoping review
Source: PLoS One. 2026 Jun 26;21(6):e0345708. doi: 10.1371/journal.pone.0345708 (PMC13308811; doi:10.1371/journal.pone.0345708)
Supplement: S1 Table — (DOCX) [file pone.0345708.s001.docx]

**Supporting information**

**S1 Table. Description of Selected Studies**

| **Level** | **Country** | **Authors and Title** | **Study Design** | **Diseases** | **Type of Integrated Disease Management** |
| --- | --- | --- | --- | --- | --- |
| Community | Australia | Hoy et al. (2005) [96]  *A chronic disease outreach program for Aboriginal communities* | Prevention program | DM  Hypertension  Renal disease | Screening |
| Community | Australia | Hoy et al. (2010) [97]  *Chronic disease profiles in remote Aboriginal settings and implications for health services planning* | Descriptive observational study | DM  Hypertension  Chronic kidney disease | Screening |
| Community | Bangladesh | Huque et al. (2018) [29] *Integrating a Diabetes and Hypertension case management package within primary health care: a mixed methods feasibility study in Bangladesh* | Mixed methods | DM  Hypertension | Guideline implementation  Staff training  Drug availability  Linkage of care |
| Community | Canada | Agarwal et al. (2019) [98] *Pilot randomized controlled trial of a complex intervention for diabetes self- management supported by volunteers, technology, and interprofessional primary health care teams* | Randomized controlled trial | DM  Hypertension | Self-assessment with mobile health application |
| Community | Canada | Harasemiw et al. (2021) [72] *Impact of point-of-care screening for hypertension, diabetes and progression of chronic kidney disease in rural Manitoba Indigenous communities* | Retrospective observational study | DM  Hypertension  Chronic Kidney disease | Screening |
| Community | China | Shi et al. (2021) [91] *Comparative effectiveness of team-based care with a clinical decision support system versus team-based care alone on cardiovascular risk reduction among patients with diabetes: Rationale and design of the D4C trial* | Cluster-randomized controlled trial | DM  HT  CVDs | Screening with integrated team-based care |
| Community | China | Ji et al. (2020) [99] *Screening for pulmonary tuberculosis in high-risk groups of diabetic patients* | Prospective cohort study  Economic evaluation | TB  DM | Screening and referral |
| Community | Ethiopia | Workneh et al. (2016a) [13] *Diabetes mellitus is associated with increased mortality during tuberculosis treatment: a prospective cohort study among tuberculosis patients in South Eastern Amahra Region, Ethiopia* | Prospective cohort study | TB  DM | Treatment |
| Community | Ethiopia | Workneh et al. (2016b) [19] *Assessment of health system challenges and opportunities for possible integration of diabetes mellitus and tuberculosis services in South-Eastern Amhara Region, Ethiopia: a qualitative study* | Qualitative study | TB  DM | Free-cost diagnostic and treatment |
| Community | Ghana | Amon et al. (2024) [100]*Household economic burden of type-2 diabetes and hypertension comorbidity care in urban-poor Ghana: a mixed methods study* | Mixed methods | DM  Hypertension | Financial costing |
| Community | India | Deo & Singh (2021) [101]*Community health worker-led, technology-enabled private sector intervention for diabetes and hypertension management among urban poor: a retrospective cohort study from large Indian metropolitan city* | Retrospective cohort study | DM  Hypertension | Screening  Treatment and monitoring by physicians |
| Community | India | Jindal et al. (2018) [92] *Development of mWellcare: an mHealth intervention for integrated management of hypertension and diabetes in low-resource settings* | Pilot feasibility study | DM  Hypertension | Mobile health application for treatment monitoring |
| Community | India | Khetan et al. (2019) [89] *Effect of a Community Health Worker-Based Approach to Integrated Cardiovascular Risk Factor Control in India A Cluster Randomized Controlled Trial* | Randomized controlled trial | DM  Hypertension  Smoking | Home-based counseling |
| Community | India | Joshi et al. (2022) [39]*Integrated Management of Diabetes and Tuberculosis in Rural India - Results from a Pilot Study* | Randomized controlled trial | TB  DM  CVD | Screening  Non-physician health workers training  Monitoring treatment adherence |
| Community | India | Koya et al. (2022) [30]*Tuberculosis and Diabetes in India: Stakeholders Perspectives on Health System Challenges and Opportunities for Integrated Care* | Qualitative outlier case study | TB  DM | Bi-directional screening  Investment in human resources, infrastructure, drug availability, and data infrastructure |
| Community | Kenya | Kasaie et al. (2020) [102]*Integrated screening and treatment services for HIV, hypertension and diabetes in Kenya: assessing the epidemiological impact and cost-effectiveness from a national and regional perspective* | Modeling study (Hybrid microstimulation and population-based model) | DM  Hypertension  HIV | Joint community outreach campaign for screening and treatment |
| Community | Kenya | Rosenberg et al. (2020) [103]*The relationship between a microfinance-based healthcare delivery platform, health insurance coverage, health screenings, and disease management in rural Western Kenya* | Cross-sectional analytical study | TB  DM  Hypertension  HIV  Cervical cancer | Screening |
| Community | Kenya | Rachlis et al. (2016) [53] *Identifying common barriers and facilitators to linkage and retention in chronic disease care in western Kenya* | Qualitative study | DM  Hypertension  HIV | Technical support  Mentorship  Training  Free-charged treatment for HIV and TB |
| Community | Kenya | Pastakia et al. (2016) [104] *Impact of Bridging Income Generation with Group Integrated Care (BIGPIC) on Hypertension and Diabetes in Rural Western Kenya* | Prospective cohort study | DM  Hypertension | Case finding  Health Promotion  Treatment |
| Community | Kenya | Vedanthan et al. (2022) [105] *Group Medical Visits and Microfinancial Intervention for Patients with Diabetes or Hypertension in Kenya* | Cluster-randomized controlled trial | DM  Hypertension | Treatment support |
| Community | Malawi | Petersen et al. (2019) [106]  *Integrated home-based screening for people living with disabilities: A case study from rural Malawi* | Case reports | TB  Hypertension  HIV  Malnutrition | Home-based screening for people living with disabilities |
| Community | Myanmar | Zayar et al. (2022) [107] Programmatic Cost-Effectiveness of a of a Second-Time Visit to  Detect New Tuberculosis and Diabetes Mellitus in TB Contact  Tracing in Myanmar | Cost analysis study | TB  DM | Contact tracing  Home-visit |
| Community | New Zealand | Hotu et al. (2010) [108] *A community-based model of care improves blood pressure control and delays progression of proteinuria, left ventricular hypertrophy and diastolic dysfunction in Mãori and Pacific patients with type 2 diabetes and chronic kidney disease: a randomized controlled trial* | Randomized controlled trial | DM  Hypertension  Chronic kidney disease | Screening |
| Community | Peru | Contreras et al. (2017) [71]  *Addressing tuberculosis patients’ medical and socio-economic needs: a comprehensive programmatic approach* | Retrospective cross-sectional study | TB  DM  HIV  Mental health | Screening of HIV, DM, and mental health among TB patients |
| Community | Peru | Millones et al. (2024) [23] *Adapting a mobile TB screening unit to provide integrated screening services and linkage to primary care* | Descriptive implementation study | TB  DM  Hypertension  Depression | Mobile TB screening unit |
| Community | Rhode Island | McAtee et al. (2024) [93]*Evaluation of a Cardiovascular Disease/Diabetes Mellitus Expansion Program for Community Health Workers Employed by Rhode Island Community Health Teams* | Quasi-experimental study | DM  HT  CVDs | Specialty trained CHW in improving patients behavior to DM-CVD |
| Community | Sierra Leone | Kachimanga et al. (2021) [75]*Implementation of a non‑communicable disease clinic in rural Sierra Leone: early experiences and lessons learned* | Retrospective observational cohort study | DM  Hypertension | Screening  Counseling  Treatment |
| Community | Singapore | Yoon et al. (2023) [7] *Socioeconomic and behavioral determinants of non-compliance with physician referrals following community screening for diabetes, hypertension and hyperlipidemia: a mixed-methods study* | Mixed methods | DM  Hypertension  Hyperlipidemia | Screening |
| Community | South Africa | Thomas et al. (2021) [90] *An analysis of the services provided by community health workers within an urban district in South Africa: a key contribution towards universal access to care* | Retrospective descriptive study | TB  DM  Hypertension  HIV | Counseling and screening by CHWs |
| Community | South Africa | Katz et al. (2009) [57]  *Managing type 2 diabetes in Soweto-The South African Chronic Disease Outreach Program experience* | Prospective cohort study | DM  Hypertension  Chronic kidney disease | Health promotion  Referral systems |
| Community | South Africa | Govindasamy et al. (2013) [59]  *Linkage to HIV, TB and Non-Communicable Disease Care from a Mobile Testing Unit in Cape Town, South Africa* | Prospective observational study | TB  DM  Hypertension  HIV | Non-physician screening for TB, DM, and HT  HIV screening, diagnosis and referral |
| Community | South Africa | Golovaty et al. (2018) [109]  *Cost of integrating non-communicable disease screening into home-based HIV testing and counseling in South Africa* | Cost analysis study | DM  HT  HIV  Dyslipidemia  Obesity  Depression  Tobacco and alcohol use | Integrated home-based screening, counseling |
| Community | South Africa | Smith et al. (2021) [110] *Reaching underserved South Africans with integrated chronic disease screening and mobile HIV counseling and testing: A retrospective, longitudinal study conducted in Cape Town* | Retrospective longitudinal study | TB  DM  HIV | Mobile health clinics for screening and counseling |
| Community | South Africa | Godongwana et al. (2021) [16]*The comorbidity of HIV, hypertension and diabetes: a qualitative study exploring the challenges faced by healthcare providers and patients in selected urban and rural health facilities where the ICDM model is implemented in South Africa* | Qualitative study | DM  Hypertension  HIV | Health promotion  Screening  Clinical management and support  Assisted self-management |
| Community | South Korea | Oh et al. (2022) [111]*Effect of an Integrative Mobile Health Intervention in Patients with Hypertension and Diabetes: Crossover Study* | Randomized controlled crossover trial | DM  Hypertension  Obesity | Mobile health approach for self-management |
| Community | UAE | Alzubaidi et al. (2019) [88]  *Diabetes and cardiovascular disease risk screening model in community pharmacies in a developing primary healthcare system: a feasibility study* | Feasibility study | DM  Hypertension  CVDs | Pharmacist-delivered screening |
| Community | Uganda | Chang et al. (2019) [56] *Challenges to hypertension and diabetes management in rural Uganda: a qualitative study with patients, village health team members, and health care professionals* | Qualitative study | DM  Hypertension | Diagnosis and treatment |
| Community | Uganda | Chamie et al. (2012) [70]  *Leveraging Rapid Community-based HIV Testing Campaigns for Non-Communicable Diseases in Rural Uganda* | Cross-sectional study | TB  DM  Hypertension  Malaria  HIV | Screening  Diagnosis  Referral  Training of counseling |
| Community | Uganda | Ingenhoff et al. (2023) [44] *Principles for task shifting hypertension and diabetes screening and referral: a qualitative study exploring patient, community health worker and healthcare professional perceptions in rural Uganda* | Qualitative study | DM  Hypertension | CHW-led community-based screening and  referral |
| Community | USA | Harvey et al. (2009) [81]  *The Healthy Connections Project: A Community-based Participatory Research Project involving women at risk of diabetes and hypertension* | Mixed methods | DM  Hypertension | Trained community health workers  Screening  Reduced behavioral factors lead to DM and HT |
| Community | USA | Abughosh et al. (2017) [112]  *A Motivational Interviewing Intervention by Pharmacy Students to Improve Medication Adherence* | Prospective quasi-experimental study | DM  Hypertension | Motivational interviewing for treatment adherence |
| Community | USA | Johnson et al. (2018) [33]  *Evaluation of an Academic Academic-Community Partnership to Implement MTM Services in Rural Communities to Improve Pharmaceutical Care for Patients with Diabetes and/or Hypertension* | Pilot implementation study | DM  Hypertension | Pharmacists’ follow-up and consultation through telephone |
| Community | USA | Lopez et al. (2024) [113] *An expanded chronic care management approach to multiple chronic conditions in Hispanics using community health workers as community extenders in the Rio Grande Valley of Texas* | Prospective longitudinal cohort study | DM  Hypertension | CHW integration in expanded chronic care model |
| Community | USA | Mohr et al. (2019) [114]*Organizational Coordination and Patient Experiences of Specialty Care Integration* | Cross-sectional study | DM  Hypertension  Mental health  CHF | Patients’ self-management with counseling and teamwork |
| Community | USA | Benzer et al. (2019) [73]*Survey of Patient-Centered Coordination of Care for Diabetes with Cardiovascular and Mental Health Comorbidities in the Department of Veterans Affairs* | Observational study | DM  Hypertension  Mental health | Patient-centered coordination of care |
| Community | USA | Ross et al. (2020) [115] *The Mississippi Delta Health Collaborative Medication Therapy Management Model: Public Health and Pharmacy Working Together to Improve Population Health in the Mississippi Delta* | Retrospective cohort study | DM  Hypertension  Dyslipidemia | Treatment |
| Community | Humanitarian settings | Ansbro et al. (2022) [35] *Chronic NCD care in crises: A qualitative study of global experts’ perspectives on models of care for hypertension and diabetes in humanitarian settings* | Qualitative study | DM  Hypertension | Morbidity prevention |
| Primary health care facility | Africa | Birungi et al. (2021) [4] *Integrating health services for HIV infection, diabetes and hypertension in sub-Saharan Africa: a cohort study* | Prospective cohort study | DM  Hypertension  HIV | Screening and follow-up treatment |
| Primary health care facility | Argentina | Belizan et al. (2020) [54]  *Barriers to hypertension and diabetes management in primary health care in Argentina: qualitative research based on a behavioral economics approach* | Qualitative study | DM  Hypertension | Systems and health care provision support for integrated disease management |
| Primary health care facility | Bangladesh | Xie et al. (2023) [40]  *Enhancing care quality and accessibility through digital technology-supported decentralisation of hypertension and diabetes management: a proof-of-concept study in rural Bangladesh* | Single-arm proof-of-concept implementation study | DM  Hypertension | Electronic medical record, linkage of care to referral, screening and follow-up |
| Primary health care | Cambodia | Chham et al. (2023) [18]  *Scaling-up integrated type-2 diabetes and hypertension care in Cambodia: what are the barriers to health system performance?* | Qualitative study | DM  Hypertension | Scaling up of integration |
| Primary health care facility | Cameroon | Labhadrt et al. (2010) [27]*Task shifting to non-physician clinicians for integrated management of hypertension and diabetes in rural Cameroon: a programme assessment at two years* | Quasi-experimental study | DM  Hypertension | Task shifting  Improvement of nurses’ knowledge |
| Primary health care facility | Canada | Provost et al. (2017) [74] *Implementation of an integrated primary care cardiometabolic risk prevention and management network in Montréal: does greater coordination of care with primary care physicians have an impact on health outcomes?* | Prospective cohort study | DM  Hypertension | Patients’ self-management, training of health workers, linkage of care, and electronic health systems |
| Primary health care facility | China | Li et al. (2012) [49]*Screening of patients with tuberculosis for diabetes mellitus in China* | Descriptive implementation study | TB  DM | Screening  Reporting |
| Primary health care facility | China | Wang et al. (2021) [5] *Pathway-Driven Coordinated Telehealth System for Management of Patients with Single or Multiple Chronic Diseases in China: System Development and Retrospective Study* | Retrospective implementation study | DM  Hypertension  COPD | Electronic medical record for both patients and health care providers |
| Primary health care facility | Eswatini | Harkare et al. (2024) [116]  *Human and financial resource needs for universal access to WHO-PEN interventions for diabetes and hypertension care in Eswatini: results from a time-and-motion and bottom-up costing study* | Time-and-motion and cost-effectiveness evaluation of WHO-PEN implementation | DM  Hypertension | Diagnosis and treatment  A paper-based Time management systems |
| Primary health care | Eswatini | Williams et al. (2023) [117]  *Diabetes–Tuberculosis Care in Eswatini: A Qualitative Study of Opportunities and Recommendations for Effective Services Integration* | Qualitative study | TB  DM | Treatment guidelines  Screening and treatment  Fast-track services |
| Primary health care facility | Ethiopia | Badacho et al. (2023) [15]  *Sustainability of integrated hypertension and diabetes with HIV care for people living with HIV at primary health care in South Ethiopia: implication for integration* | Cross-sectional descriptive study | DM  Hypertension  HIV | Integration of service in HIV, DM, and hypertension |
| Primary health care | Ethiopia | Badacho et al. (2024) [63]  *Lived experiences of people living with HIV and hypertension or diabetes access to care in Ethiopia: a phenomenological study* | Qualitative study | DM  Hypertension  HIV | Treatment of PLWH with comorbid |
| Primary health care facility | Ethiopia | Mulugeta et al. (2022) [86]  *Readiness of the primary health care units and associated factors for the management of hypertension and type II diabetes mellitus in Sidama, Ethiopia* | Cross-sectional study | DM  Hypertension | Preparation of drugs, diagnostic equipment, and place for clinic, and staff training |
| Primary health care facility | Ghana | Salifu & Hlongwana (2021) [42]  *Exploring the mechanisms of collaboration between the Tuberculosis and Diabetes Programs for the control of TB-DM Comorbidity in Ghana* | Qualitative descriptive study | TB  DM | Collaboration between TB and NCD programs in Ghana at the levels of national, regional and health facilities |
| Primary health care facility | Ghana | Salifu & Hlongwana (2021) [79]  *Barriers and facilitators to bidirectional screening of TB-DM in Ghana: Healthcare workers’ perspectives* | Qualitative study | TB  DM | Bidirectional screening |
| Primary health care facility | India | Jayanna et al. (2019) [46] *Designing a comprehensive Non- Communicable Diseases (NCD) programme for hypertension and diabetes at primary health care level: evidence and experience from urban Karnataka, South India* | Mixed methods | DM  Hypertension | Health promotion, screening, and treatment  Public and private partnership |
| Primary health care facility | India | Naik et al. (2013) [80]*Is screening for diabetes among tuberculosis patients feasible at the field level?* | Descriptive cross-sectional study | DM  TB | Free-cost screening |
| Primary health care facility | India | Majumdar et al. (2013) [62]*Tuberculosis-diabetes screening: how well are we doing? A mixed-methods study from North India* | Mixed methods | DM  TB | Screening |
| Primary health care | India | Patel et al. (2020) [69] *The Integrated Tracking, Referral, and Electronic Decision Support, and Care Coordination (I-TREC) program: scalable strategies for the management of hypertension and diabetes within the government healthcare system of India* | Quasi-experimental study | DM  Hypertension | Electronic medical records integration patients’ tracking, referral system, and coordination of care |
| Primary health care facility | India | Raghuveer et al. (2020) [118]  *Opportunistic screening for diabetes mellitus and hypertension in primary care settings in Karnataka, India: a few steps forward but still some way to go* | Mixed methods | DM  Hypertension | Opportunistic screening by laborian |
| Primary health care facility | Indonesia | Arini et al. (2022) [119] *Challenges, opportunities, and potential roles of the private primary care providers in tuberculosis and diabetes mellitus collaborative care and control: a qualitative study* | Qualitative study | TB  DM | Integrated Screening  Health promotion  Diagnosis  Treatment |
| Primary health care facility | Iran | Soleimani et al. (2024) [120] *Self-management education for hypertension, diabetes, and dyslipidemia as major risk factors for cardiovascular disease: Insights from stakeholders’ experiences and expectations* | Qualitative study | DM  Hypertension  Dyslipidemia | Self-management education |
| Primary health care facility | Kenya | Some et al. (2016) [77] *Task Shifting the Management of Non-Communicable Diseases to Nurses in Kibera, Kenya: Does it Work?* | Descriptive retrospective observational study | DM  Hypertension  Epilepsy  Asthma  Sickle cell | Task shifting to nurses |
| Primary health care | Kenya | Otieno et al. (2024) [26]  *Perceived health system facilitators and barriers to integrated management of hypertension and type 2 diabetes in Kenya: a qualitative study* | Qualitative study | DM  Hypertension | One stop centre screening, diagnosis, and treatment |
| Primary health care facility | Lebanon | Kayali et al. (2019) [121]  *Treating Syrian refugees with diabetes and hypertension in Shatila refugee camp, Lebanon: Médecins Sans Frontières model of care and treatment outcomes* | Descriptive retrospective cohort study | DM  Hypertension | Screening, counseling, and health promotion |
| Primary health care facility | Lebanon | Saleh et al. (2018) [67]  *Using Mobile Health to Enhance Outcomes of Noncommunicable Diseases Care in Rural Settings and Refugee Camps: Randomized Controlled Trial* | Randomized controlled trial | DM  Hypertension | Mobile health application |
| Primary health care facility | Malawi | Nyirenda et al. (2022) [21]  *Bidirectional screening and treatment outcomes of diabetes mellitus (DM) and tuberculosis (TB) patients in hospitals with measure to integrate care of DM and TB and those without integration measures in Malawi* | Retrospective cohort study | TB  DM | Screening and treatment |
| Primary health care | Malawi | Pence et al. (2024) [45]  *Two implementation strategies to support the integration of depression screening and treatment into hypertension and diabetes care in Malawi (SHARP): parallel, cluster-randomised, controlled, implementation trial* | Cluster-randomized controlled trial | DM  Hypertension  Depression | Screening of mental health  Algorithm-guided of screening |
| Primary health care facility | Malawi | Wroe et al. (2019) [34]  *Expanding access to non-communicable disease care in rural Malawi: outcomes from a retrospective cohort in an integrated NCD–HIV model* | Retrospective cohort study | DM  Hypertension  HIV  Epilepsy  Asthma  Mental health | Healthcare referral and patient’s follow-up |
| Primary health care facility | Malaysia | Ariffin et al. (2017) [122]  *Feasibility of Implementing Chronic Care Model in the Malaysian Public Primary Care Setting* | Cross-sectional descriptive study | DM  Hypertension | Strengthening of health workers and training |
| Primary health care facility | Malaysia | Mohd et al. (2023) [87]  *Innovative Integrated Motivational Interviewing for Dual Management in Tuberculosis Patients with Diabetes (MID-DOT) in Malaysia* | Randomized-controlled trial study | TB  DM | Motivational interviewing and direct observed treatment |
| Primary health care | Nepal | Kumar et al. (2019) [95]  *Designing and implementing an integrated non-communicable disease primary care intervention in rural Nepal* | Retrospective cohort implementation study | DM  Hypertension  COPD | Health workers training, electronic medical record, counseling |
| Primary health care facility | Pakistan | Basir et al. (2019) [48] *Operationalization of bi-directional screening for tuberculosis and diabetes in private sector healthcare clinics in Karachi, Pakistan* | Implementation study | TB  DM | Bidirectional screening |
| Primary health care facility | Philippines | Hashiguchi et al. (2023) [83]  *How can tuberculosis services better support patients with a diabetes co-morbidity? A mixed methods study in the Philippines* | Mixed methods | TB  DM | Screening  Diagnosis and treatment |
| Primary health care facility | Sierra Leone | Zou et al. (2020) [9]  *Adapting and implementing training, guidelines and treatment cards to improve primary care-based hypertension and diabetes management in a fragile context: results of a feasibility study in Sierra Leone* | Feasibility study | DM  Hypertension | Guideline implementation  Health workers training |
| Primary health care facility | Singapore | Hu et al. (2023) [123]  *Integrated care teams in primary care improve clinical outcomes and care processes in patients with non-communicable diseases* | Retrospective cohort study | DM  Hypertension  Hyperlipidemia | Electronic medical records |
| Primary health care facility | Slovenia | Stojnić et al. (2023) [25]  *Perceptions of the primary health care team about the implementation of integrated care of patients with type 2 diabetes and hypertension in Slovenia: qualitative study* | Qualitative study | DM  Hypertension | Integration of diagnosis, treatment of care |
| Primary health care facility | South Africa | Murudi-Manganye et al. (2022) [124] *An Integrated Reporting Tool for Management of HIV And Non-communicable Diseases for Primary Health Care Facilities in Limpopo Province, South Africa* | Tool development and validation study | DM  Hypertension  HIV | Recording and reporting |
| Primary health care | South Africa | Pinxteren et al. (2023) [52]  *The impact of persistent precarity on patients’ capacity to manage their treatment burden: A comparative qualitative study between urban and rural patients with multimorbidity in South Africa* | Qualitative study | TB  DM  Hypertension  HIV | Counseling and treatment |
| Primary health care facility | South Asia | Shah et al. (2012) [76] *Improving diabetes care: Multi-component cardiovascular disease risk reduction strategies for people with diabetes in South Asia–The CARRSMulti-center Translation Trial* | Randomized controlled trial | DM  HT  Dyslipidemia | Guideline implementation, electronic medical records, feedback assessment on cost of health services |
| Primary health care facility | Spain | Etxeberria et al. (2018) [94] *Results from the CLUES study: a cluster randomized trial for the evaluation of cardiovascular guideline implementation in primary care in Spain* | Cluster-randomized controlled trial | DM  Hypertension | Guideline implementation, staff training, electronic health systems for treatment algorithm |
| Primary health care facility | Tanzania | Miselli et al. (2021) [41]  *An Integrated Management System for Noncommunicable Diseases Program Implementation in a Sub-Saharan Setting* | Implementation study | DM  Hypertension | Screening, physical assessment by specialists, and free services of follow-up |
| Primary health care facility | Tanzania | Mpagama et al. (2023) [10]  *Implementing Innovative Approaches to Improve Health Care Delivery Systems for Integrating Communicable and Non-Communicable Diseases Using Tuberculosis and Diabetes as a Model in Tanzania* | Implementation study | TB  DM | Integration of service in DM and TB  Bidirectional screening  Web-based training |
| Primary health care facility | Tanzania | Shayo et al. (2022) [12]  *The acceptability of integrated healthcare services for HIV and non-communicable diseases: experiences from patients and healthcare workers in Tanzania* | Qualitative study | DM  Hypertension  HIV | One-stop centre clinic |
| Primary health care facility | Uganda | Bukenya et al. (2022) [84]  *Integrated healthcare services for HIV, diabetes mellitus and hypertension in selected health facilities in Kampala and Wakiso districts, Uganda: A qualitative methods study* | Qualitative study | DM  Hypertension  HIV | Screening and assessment through one clinic |
| Primary health care facility | Uganda | Moor et al. (2022) [125]  *Patient preferences for facility-based management of hypertension and diabetes in rural Uganda: a discrete choice experiment* | Discrete choice experiment (DCE) | DM  Hypertension | Facility based management in rural area |
| Primary health care facility | Uganda | Muwanguzi et al. (2023) [17]  *Barriers and facilitators to cognitive impairment screening among older adults with diabetes mellitus and hypertension by primary healthcare providers in rural Uganda* | Descriptive qualitative study | DM  Hypertension  Cognitive impairment | Screening  Cognitive impairment screening |
| Primary health care faclity | Uganda | Sando et al. (2020) [126] *Cost‐effectiveness analysis of integrating screening and treatment of selected non‐communicable diseases into HIV/AIDS treatment in Uganda* | Cost analysis study | DM  Hypertension  Hypercholesterolemia  HIV/AIDS | Screening |
| Primary health care | Uganda | Tusubira et al. (2023) [68]  *Self-care and healthcare seeking practices among patients with hypertension and diabetes in rural Uganda* | Cross-sectional study | DM  Hypertension | Patients’ self-management |
| Primary health care facility | Uganda | Tusubira et al. (2021) [2]  *Social Support for Self-Care: Patients Strategies for Managing Diabetes and Hypertension in Rural Uganda* | Cross-sectional qualitative study | DM  Hypertension | Screening, counseling, and follow-up |
| Primary health care facility | UK | Istepanian et al. (2009) [127]*Technical and Compliance Considerations for Mobile Health Self-monitoring of Glucose and Blood Pressure for patients with* *Diabetes* | Randomized controlled trial | DM  Hypertension | Mobile health approach for self-management |
| Primary health care facility | USA | Brunisholz et al. (2018) [128]  *“Pharming Out” support: a promising approach to integrating clinical pharmacists into established primary care medical home practices* | Retrospective cohort study | DM  Hypertension | Collaboration of pharmacists and physicians for home visit during treatment |
| Primary health care facility | USA | Coe et al. (2020) [129] *Pharmacists providing care in statewide physician organizations: findings from the Michigan Pharmacists Transforming Care and Quality Collaborative* | Descriptive observational study | DM  Hypertension  Hyperlipidemia | Collaborative care between pharmacist and physician |
| Primary health care facility | USA | Roll et al. (2020) [130]  *The design and evaluation of a pilot covisit model: Integration of a pharmacist into a primary care team* | Quasi-experimental study | DM  Hypertension | Pharmacist and primary care team collaboration |
| Primary health care facility | Vietnam | Quynh-Anh et al. (2024) [131]  *Continuity of primary care for type 2 diabetes and hypertension and its association with health outcomes and disease control: insights from Central Vietnam* | Cross-sectional study | DM  Hypertension | Patients’ follow-up by health workers within regular visits |
| Referral | Angola | Segafredo et al., (2019) [31]*Integrating TB and non-communicable diseases services: Pilot experience of screening for diabetes and hypertension in patients with Tuberculosis in Luanda, Angola* | Cross-sectional study | TB  DM  Hypertension | Integrated screening for all newly diagnosed pulmonary TB |
| Referral | Australia | Katz et al., (2018) [3]*iConnect CKD - virtual medical consulting: A web-based chronic kidney disease, hypertension and diabetes integrated care program* | Randomized-controlled trial | DM  Hypertension  Chronic kidney disease | Web-based integrated care program |
| Referral | China | Tu et al. (2020) [1]*A transitional care intervention for hypertension control for older people with diabetes: a cluster randomized controlled trial* | Cluster-randomized controlled trial | DM  Hypertension | Self-management education  Lifestyle changes  Individualized medication  Primary care visits  Telephone support |
| Referral | China | Xiao et al., (2021) [60]*Delayed diagnosis of tuberculosis in patients with diabetes mellitus co-morbidity and its associated factors in Zhejiang Province, China* | Retrospective cross-sectional study | TB  DM | Diagnosis |
| Referral | Ethiopia | Jerenea et al., (2017) [51]*The yield and feasibility of integrated screening for TB, diabetes and HIV in four public hospitals in Ethiopia* | Feasibility study | TB  DM  HIV | Integrated screening |
| Referral | Ethiopia | Nunemo, et al (2023) [20]*Integration Challenges and Opportunity of Implementing Non-Communicable Disease Screening Intervention with Tuberculosis Patient Care: A Mixed Implementation Study* | Mixed implementation study | TB  DM  Hypertension | Integrated screening |
| Referral | Ghana | Owusu et al. (2024) [24]*A qualitative exploration of policy interventions to improve the health-related quality of life of people living with HIV AIDS and co-morbidities of hypertension and/or diabetes in Ghana* | Qualitative study | DM  Hypertension  HIV | Support groups  Home visits  Provision of free drugs  Counseling |
| Referral | India | Anand et al., (2018) [65]*Integrating screening for non-communicable diseases and their risk factors in routine tuberculosis care in Delhi, India: A mixed methods study* | Mixed methods | TB  DM  Hypertension | Integrated screening among TB patients |
| Referral | India | Kumar et al. (2013) [58]*Screening of patients with diabetes mellitus for tuberculosis in India* | Prospective operational feasibility study | TB  DM | Screening |
| Referral | Indonesia | Ruslami et al. (2021) [37]*The effect of a structured clinical algorithm on glycemic control in patients with combined tuberculosis and diabetes in Indonesia: A randomized trial* | Randomized-controlled trial | TB  DM | Counseling, monitoring, and treatment |
| Referral | Kenya | Oyugi et al. (2020) [132]*Improving the management of hypertension and diabetes: An implementation evaluation of an electronic medical record system in Nairobi County, Kenya* | Implementation study | DM  Hypertension | Integrated electronic medical records |
| Referral | Malawi | Kachimanga et al., (2017) [36]*Novel approaches to screening for noncommunicable diseases: Lessons from Neno, Malawi* | Descriptive study | DM  Hypertension  HIV | Integrated screening |
| Referral | Southern Malawi | Katundu et al. (2024) [22]*Barriers and facilitators to integration of screening for hypertension, diabetes mellitus and dyslipidemia, among adult people living with HIV at district hospital ART clinics in Southern Malawi* | Mixed methods | DM  Hypertension  Dyslipidemia  HIV | Screening  Integration of electronic medical records  Screening by health community workers  Partnership with NGOs |
| Referral | Tanzania | Bintabara & Ngajilo (2020) [11]*Readiness of health facilities for the outpatient management of noncommunicable diseases in a low resource setting: an example from a facility-based cross-sectional survey in Tanzania* | Facility-based cross-sectional survey | DM  Hypertension  Chronic respiratory diseases | Staff training and guideline  Diagnosis  Treatment |
| Referral | USA | Marquard et al. (2013) [82]*Overcoming challenges integrating patient-generated data into the clinical EHR: Lessons from the CONtrolling Disease Using Inexpensive IT-Hypertension in Diabetes (CONDUIT-HID) Project* | Developmental and implementation process evaluation study | DM  Hypertension | Integration of patients’ BP data into electronic medical records |
| Referral | USA | Price-Haywood et al. (2017) [50]*Clinical Pharmacist Team-Based Care* *in a Safety Net Medical Home:* *Facilitators and Barriers to Chronic Care Management* | Prospective observational implementation study | DM  Hypertension | Pharmacists for counseling of newly diagnosed patients with DM and hypertension |
| Referral | USA | Wong et al. (2013) [28]*Impact of care management processes and integration of care on blood pressure control in diabetes* | Cross-sectional multisite observational study | DM  Hypertension | Patients’ self-management support through reminder  Screening for hypertension  Linkage to referral system  Guideline implementation |
| Referral | Zimbabwe | Frieden et al., (2020) [14]*Setting up a nurse-led model of care for management of hypertension and diabetes mellitus in a high HIV prevalence context in rural Zimbabwe: a descriptive study* | Qualitative study | DM  Hypertension | Nurse-led screening in rural area |
| Community  Primary health care facility Referral | India | Jindal et al. (2022) [78]*Improving care for hypertension and diabetes in India by addition of clinical decision support system and task shifting in the national NCD program: I-TREC model of care* | Pilot implementation study | DM  Hypertension | Integrated screening within all level of care |
| Community  Primary health care facility Referral | Ghana | Salifu et al. (2021) [133]*Frontline healthcare workers’ experiences in implementing the TB-DM collaborative framework in Northern Ghana* | Qualitative study | TB  DM | Bi-directional screening  TB and HIV monitoring system |
| Primary health care  Referral | India | Rupani & Vyas. (2023) [61] *A sequential explanatory mixed-methods study on costs incurred by patients with tuberculosis comorbid with diabetes in Bhavnagar, western India* | Mixed methods | TB  DM | Screening  Diagnosis  Treatment  Counseling  Cash assistance  Home-delivered care |
| Primary health care facility  Referral | Malawi | Pfaff et al. (2017) [47]  *You can treat my HIV - But can you treat my blood pressure? Availability of integrated HIV and noncommunicable disease care in northern Malawi* | Cross-sectional study | DM  Hypertension  HIV | Screening  Consultation |
| Primary health care facility  Referral | Uganda | Tusubira et al. (2020) [134] *Accessing medicines for non-communicable diseases: Patients and health care workers’ experiences at public and private health facilities in Uganda* | Qualitative study | DM  Hypertension | Treatment |
| Primary health care facility  Referral | Tanzania | Chamba et al. (2022) [135]  *Where can Tanzania health system integrate clinical management of patients with dual tuberculosis and diabetes mellitus? A cross-sectional survey at varying levels of health facilities* | Cross-sectional study | TB  DM | Guideline implementation  Trained health workers  Accessibility of diagnostic equipment |
| Primary health care  Referral | Tanzania | Ottaru et al. (2024) [64]  *“I only seek treatment when I am ill”: experiences of hypertension and diabetes care among adults living with HIV in urban Tanzania* | Qualitative study | DM  Hypertension  HIV | Free-cost treatment |
