# Supplementary material for: Potentials, barriers, and strategies for integrating tuberculosis, diabetes mellitus, and hypertension case management: A scoping review
Source: PLoS One. 2026 Jun 26;21(6):e0345708. doi: 10.1371/journal.pone.0345708 (PMC13308811; doi:10.1371/journal.pone.0345708)
Supplement: S2 Table — (DOCX) [file pone.0345708.s002.docx]

**Supporting information**

**S2 Table. Barriers and Key Strategies of Integrating TB, DM, and hypertension management**

|  | **Barriers** | **Key strategies** |
| --- | --- | --- |
| **Leadership and Governance** | **System and Strategies** | |
|  | - Unclear strategies from government and lacking supports and leadership from relevant organizations and poor leadership [23,48,49,52,71,78-83] - Lack of system to monitor and evaluate the integration [49,55,71,78-80] | - Creation of TB/DM working group at national level [22,79] - Cooperate with supporting organization of key affected populations [23,79,80] and involve community leaders [33,39,84] - Collaborate with local stakeholders and non-medical professionals such as pharmacists [29,33,51,64,83], non-medical professionals [27,31,33,67,70,73,83-85], community [19,33,49,81,83,86-88] - Decentralization of health services/program [49,52,79,89,90] - Embedding the program into a national NCD strategy [83,91] |
|  | **Guidelines** | |
|  | - Inadequate guidelines [52,55,70,78,83,92-94] - Social health insurance mismatched regulations [95] | Develop management guidelines [70,82,92,96], adequate guideline implementation [43,53-55,60,61,79,82,84,88,91,97-100], and making simplified clinical guidelines [52] |
| **Health service delivery** | **System of Delivery** | |
|  | - Suboptimal guideline implementation [56,82,94,102] - Poor clinic health systems [84,103] | - Algorithm-based treatment [28,60,100] - Individualized medication treatment plan [30,33,40,48,65,83,85,104] - Clinical decision support system [30,60,69] - Patient centered care [33,40,45,61,77] - Use standardized protocols [52,69,85,88] |
|  | **Time of Delivery** | |
|  | - Long waiting time [21,30,82,89] - Multiple consultation of patients [2] - Time consuming [15,34,46,42,49,81,82,95,102,105,106] - Multiple appointments needed [82] - Reminders were not delivered to the intended patients | - Simple and short screening tools [21,33,48,85,107] - Integrating services [16,18,22,24-27,30,33,35,40,48-50,53,65,82,85,87-89,107,108] - Virtual consultation with GP [3] - Mobile health clinics [38,101] - Provide time after-hours and weekend clinics [42] - Home visit [15,83,109-111] |
|  | **Screening** | |
|  | - Absence of early detection and disease management system [14,23,49,88,106] - Delays in screening [91] | - Community-based approach [84,112] - Home-based screening [33,37,67,113] - Targeted screening programs [35,114] - Identifying needs of the people with difficult access to screening [37] - Combined with a routine chronic disease program [96] - Mental health screening in patients with HIV and NCDs [85,115] - Point-of-care testing [114] - Develop screening model [116] - TB screening in high-risk DM patients [117,118] |
|  | **Service Delivery** | |
|  | - Limited awareness of collaborative framework [18,23,84,88,89,95,105] - Separated disease management [14,23,91,105,106] - Lack of practical solution and management [30,45] - Expensive partnership with private sector [51,84] - Limited DM service [79] | - Referral to higher health facilities [28,42,49-51,55,62,65,77,83,99,110,114,119-121] - System of tracking loss to follow up patients [2,4–8] - Patient’s self-management [14,30,32,33,40,45,48,59,62,73,75,77,90,108,122,123] - Two-way referral [30,33,40-50] - Active case finding [38,42,67,82,119,124] - Post-discharge support from community health centers [1] - Link intervention and treatment in peer groups [43,50] - Chronic disease outreach program [15,103] - Mobile phone application to record lifestyle, self-measure, and provide feedback and health information [123] - Group medical visits with a community health worker and clinician [125] |
|  | **Context of Service** | |
|  | - Perceived less effective medication when the treatments are combined [14,30,84,108] - Less information about the comorbidities of diseases [14,30,121] - Less physical or dietary intervention [49,121,126] - Unwillingness of patients [59,82] - Stigma of patients from others [81-83,89,91,108] | - Counseling and education [18,21,28,31-33,40,42,45-47,50,51,53-55,57,64,67,71,72,82,83,85,90,97,109,119,120,126-128] - Health Education [17,33,40-50] - Tailoring educational interventions [45] |
|  | **Referral Services** | |
|  | - Lack of access and financial support to go to referral care [18,24-26,29,30,52,65,71,81,83,84,94,102,121,127,129] - Loss of referral letters [42] - Retention by specialist [50,102] | - Mobile health application for reminder [32,54,60,63] - Mobile health clinics [38,101] - Coordination of care involving specialists [65,76] |
| **Health workforce** | **Training** | |
|  | - Short training duration [9] - Limited competency of healthcare workers due to insufficient training [21,23,30,49,52,61,66,79-81,88,91,103,130,131] | - Adequate training of health workers or non-health workers [16,19,21,23,24,26,27,29,31,33,36,39,41,44,46,49,51,53,54,56,62-64,69,71,72,82,83,87,88,93,95,99-101,103,113,116,119,124,126,129,131-134] - Training using web-based [10] - Continuous accessible training [49,102] |
|  | **Health Workers** | |
|  | - Short staffed [27,52,53,60,72,79-81,84,88,95,96,102,103,105,112,120,127] - Lack of health worker’s adherence to testing and treatment protocols [26,82,88,112,135] - High staff turnover [52,83,99,103,127] - Absenteeism of the employee [74,112] - Unwillingness of health workers [30,84] - Overconfidence of health workers [30,102] | - Continued support from community health workers [33,39,44,46,47,50,79,80,82-84,87,96,112,114,135] - Linkage to associations [49] - Hire additional temporary staff [53] |
|  | **Workforce** | |
|  | - High burden of works [21,23,30,49,52,61,66,79-81,88,91,103,105,131] | - Task-shifting [19,39,50,53,60,79,80,91,98,126,131,134,135] |
|  | **Collaboration** | |
|  | - Lack of interprofessional collaboration [52,61,73,103] | - Interprofessional collaboration with dieticians, nurse, and/or pharmacist, and counselors [33,40,49,52,54,68,70,82,84,104,116,128,136] |
|  | **Guideline** | |
|  | - Risk of infection [80] - Not prioritizing counseling [14,102] | - Context-specific and easy-to-use guideline for CHW [9] |
| **Health information systems** | **Recording and Reporting** | |
|  | - Separated report systems [16,23,49,105,128] - Treatment cards incompletely filled [29,105] - No individual patient record [55] - Poor recording and reporting systems [63,70,71,75,95,100] | - Parallel registration: integrated medical history [25,71] - Using electronic medical record [19,28,32,33,48,51-65] - Electronic application to facilitate guidelines-based assessment and management [44] - Integrated technology platform to assess patients’ risk level and provide recommendations [19,83,92] - Integrated medical records consist of medical guidelines, health insurance plans, and medicine availability [69] |
|  | **Data management** | |
|  | - Poor data management [16,49,79,84,105] - Difficult retrieval of data collection and cross-verification [105] | - Digitized monitoring system [48,51,65,71,74] |
|  | - Difficulty in operating electronic health records [32,50,59,137] - Limitations in electronic medical record functions [48,52] | - Treatment cards [29,61,90] |
| **Medical products, vaccines, and technologies** | - Shortages of medication and equipment to evaluate [14,18,21,23,29,30,41,51,53,79-84,86,89,93,95,96,99,102,103,127] - Diagnostic equipment not available [51,83,88] - High cost of drugs [14,41,57,79,80,130] - Expensive technologies [48] - Lack of medicine request procedure [16,84] - Not common drugs and low quality of drugs [2] | - Provide medical equipment needed [41,53,80,134] - Provide medicine needed [14,29,80,83,108,134,138] - Integrated collectible drug site [66,108,115,132] - Supported and subsidized by health authorities and/or NGOs [33,85,134] - Website for healthy lifestyle promotion [49] |
| **Health system financing** | **Financial** | |
|  | - Patient's financial constraints [15,21,29,30,79,83,89,91,108,127,129] - Financial constraints [23,26,49,53,57,65,79-84,89,91,92,96,129,130,135,136,139] - Selection of patients given drugs because of financial constraints [30] | - Free-charged service [15,17,24,30,42,44,53,80,83,102,107,119,129,138] - Financial support from organization [33,52,81,84,127] - Generate demand for care through incentives [43,108] - Financial supports from family and friend [14,139] |
|  | **Insurance** | |
|  | - Lack of insurance coverage [50,81-83,89,108,139] | - Community insurance [33,79,80,84,96] - Available financial protection [44,66,80] |
|  | **Budgeting System** | |
|  | - Unstandardized budget allocations in each health care [84] | - More cost-effective when the disease management integrated [42,53,79,140] - Prioritize essential NCD interventions [66] - Microfinance program integrated with healthcare delivery [78,125] |
|  | - Delays in the distribution of allocated funds [93] - Affected adherence to medication due to financial problems [30,139] |  |
